# Supplementary material for: Predicting mortality in geriatric patients with peptic ulcer bleeding: a retrospective comparative study of four scoring systems
Source: PeerJ. 2025 Mar 17;13:e19090. doi: 10.7717/peerj.19090 (PMC11925040; doi:10.7717/peerj.19090)
Supplement: Supplemental Information 2 [file peerj-13-19090-s002.docx]

Codebook for RAW data:

Sex: 1-Male; 0-Female

Altered Mental Status: 0-absent; 1-present

Acute coronary syndrome: 0-absent; 1-present

Congestive Heart Failure: 0-absent; 1-present

Peripheral Vascular Disease: 0-absent; 1-present

Cerebrovascular Disease: 0-absent; 1-present

Dementia: 0-absent; 1-present

Hemiplegia or paraplegia: 0-absent; 1-present

Chronic pulmonary disease: 0-absent; 1-present

Rheumotologic disease: 0-absent; 1-present

Peptic Ulcer Disease: 0-absent; 1-present

Diabetes Mellitus: 0-absent; 1-present

Chronic Kidney Disease: 0-absent; 1-present

Liver Disease: 0-absent; 1-present

Malignity: 0-absent; 1-present

Localised solid tumor: 0-absent; 1-present

Leukomia or lymphoma: 0-absent; 1-present

Metastatic solid tumour: 0-absent; 1-present

Syncope: 0-absent; 1-present

Melena: 0-absent; 1-present

Vomiting fresh blood: 0-absent; 1-present

Steroids: 0-absent; 1-present

Anticoagulants: 0-absent; 1-present

Antiplatelet: 0-absent; 1-present

NSAIDs: 0-absent; 1-present

Cause of bleed: 1-gastric ulcer; 2-dudenal ulcer; 3-others

Rebleeding: 0-absent; 1-present

In-hospital mortality: 0-absent; 1-present
